# Exploring the Potentials of Artificial Intelligence in Sepsis Management in the Intensive Care Unit

**DOI:** 10.1155/ccrp/9031137

**Published:** 2025-08-28

**Authors:** Ali Riahi, Mohammad Sepehr Yazdani, Reza Eshraghi, Motahare Karimi Houyeh, Ashkan Bahrami, Sara Khoshdooz, Mahshid Amini, Ehsan Behzadi, Amirreza Khalaji, Seyed Masoud Moeini Taba, Seyed Mohammad Reza Hashemian

**Affiliations:** ^1^School of Medicine, Isfahan University of Medical Sciences, Isfahan, Iran; ^2^Student Research Committee, Kashan University of Medical Sciences, Kashan, Iran; ^3^Social Determinants of Health Research Center, Isfahan University of Medical Sciences, Isfahan, Iran; ^4^Faculty of Medicine, Guilan University of Medical Sciences, Rasht, Iran; ^5^Department of Orthopedic Surgery, Kashan University of Medical Sciences, Kashan, Iran; ^6^Faculty of Medicine, Tabriz University of Medical Sciences, Tabriz, Iran; ^7^Department of Nephrology, Kashan University of Medical Sciences, Kashan, Iran; ^8^Clinical Tuberculosis and Epidemiology Research Center, National Research Institute of Tuberculosis and Lung Diseases (NRITLD), Shahid Beheshti University of Medical Sciences, Tehran, Iran

**Keywords:** artificial intelligence, deep learning, ICU, machine learning, sepsis diagnosis

## Abstract

Sepsis remains one of the leading causes of morbidity and mortality worldwide, particularly among critically ill patients in intensive care units (ICUs). Traditional diagnostic approaches, such as the Sequential Organ Failure Assessment (SOFA) and systemic inflammatory response syndrome (SIRS) criteria, often detect sepsis after significant organ dysfunction has occurred, limiting the potential for early intervention. In this study, we reviewed how artificial intelligence (AI)–driven methodologies, including machine learning (ML), deep learning (DL), and natural language processing (NLP), can aid physicians. AI, in this case, particularly ML, processes massive amounts of real-time clinical data, vital signs, lab results, and patient history and can detect subtle patterns and predict sepsis earlier than traditional methods like SOFA or SIRS, which often lag behind after the presentation of the sequela. Models like random forest, XGBoost, and neural networks achieve high accuracy and area under the receiver operating characteristic curve (AUROC) scores (0.8–0.99) in ICU and emergency settings, enabling timely intervention by distinguishing sepsis from similar conditions despite the lack of perfect biomarkers. In practice, however, there are several potential pitfalls. Algorithmic bias due to nonrepresentative data, data fragmentation, lack of validation, and explainability issues are current barriers in developed models. Future research should address these limitations and develop more sophisticated models.

## 1. Introduction

Sepsis is a life-threatening organ malfunction that is a consequence of an abnormal host response to infection [1]. In 2017, an estimated 48.9 million incident cases of sepsis and 11 million sepsis-related deaths were reported worldwide, accounting for 19.7% of all deaths [2]. In the intensive care unit (ICU) where the patients are experiencing a critical situation, the probability of developing sepsis is even greater and even perilous, attributed to dysregulated immune response [3]. In a meta-analysis study of patients with sepsis and organ dysfunction, 24.4% of cases (ranging between 10.3% and 42.5%) acquired sepsis during ICU stay, and 48.7% (ranging between 18.7% and 69.4%) acquired sepsis from a hospital origin [4].

The Sepsis-1 definition, which predates the current definition, was developed at a consensus conference in 1991. In that definition, sepsis is characterized by the presence of two or more criteria of systemic inflammatory response syndrome (SIRS) alongside an established or suspected infection [5]. Currently, the Sequential Organ Failure Assessment (SOFA) scoring system is used to aid sepsis diagnosis. The SOFA score evaluates a patient's organ function across six systems [1]. These diagnostic scores, however, are more reactive than predictive, often diagnosing sepsis only after significant organ damage has occurred [6]. In addition, several biomarkers, including C-reactive protein (CRP), procalcitonin, and white blood cell (WBC) count, are used in sepsis diagnosis [7]. While these markers are helpful, they can be elevated in nonspecific, nonseptic inflammatory conditions such as trauma or surgery, resulting in insufficient sensitivity to reliably rule sepsis in or out [8, 9]. It seems that the best protocol for timely and accurate detection of sepsis is a combination of all influential diagnostic factors, suggesting a need for enhanced methods, potentially developed by incorporating advanced technologies like nanodiagnostics and machine learning (ML) models [10, 11].

In addition to the challenges in detection, the management of sepsis patients, particularly those experiencing septic shock, faces various challenges. The heterogeneity of patients' characteristics, encompassing immunity status, organ functions, and underlying comorbidities, is a significant obstacle to sepsis management [12]. Additionally, varying causative agents, rampant antimicrobial resistance (AMR), and under-resourced healthcare systems complicate management [13]. Regarding these obstacles, artificial intelligence (AI) can help us surmount them. It can gather and analyze all patients' characteristics, laboratory findings, and comorbidities, and help predict AMR [14, 15], determine the most appropriate antibiotic [14, 16], and even contribute to the development of novel antimicrobial drugs on a broader scale [17]. Steinbach et al. (2024) developed and externally validated a robust ML model that relies solely on complete blood count (CBC) parameters, combined with patient demographics, to predict sepsis requiring ICU admission with high accuracy (area under the receiver operating characteristic curve (AUROC) up to 0.872) [18]. This study underscores the feasibility of integrating easily accessible laboratory data into clinical decision support systems (CDSSs) to enable timely and cost-effective sepsis diagnosis, particularly in resource-limited environments where complex vital sign monitoring may be unavailable. However, a comprehensive review analyzed 39 studies on ML models using laboratory data for sepsis diagnosis and prognosis, highlighting substantial heterogeneity in study populations, sepsis criteria, features used, and validation methods [19]. For instance, Persson et al. (2024) conducted the largest prospective randomized clinical validation of a ML-based sepsis prediction algorithm, NAVOY® Sepsis, in an ICU setting [20]. Their algorithm demonstrated robust prognostic accuracy, predicting sepsis onset 3 h in advance with an accuracy of 79%, sensitivity of 80%, specificity of 78%, and an AUROC of 0.80. Importantly, the algorithm maintained comparable performance in patients with COVID-19, despite being primarily trained on bacterial sepsis cases [20]. Nonetheless, as shown by AUROC and accuracy values, the performance of the current models should be elevated.

In this study, we review recent studies that applied AI-based approaches to the management of sepsis in the ICU, focusing on improving early prediction and diagnosis, optimizing antibiotic therapy, enhancing monitoring and alert systems, and advancing personalized medicine (PM). In addition to reviewing the performance rates, we highlighted the barriers to employing AI systems and proposed potential solutions.

## 2. AI in Healthcare: An Overview

AI is a technological science focused on developing theories, methods, and applications to simulate and enhance human intelligence [21–23]. ML and deep learning (DL) are cutting-edge disciplines in computer science [24]. ML involves training computers to learn from historical data to predict future outcomes or behaviors, while DL, a subset of ML, utilizes artificial neural networks to train and analyze data, often handling complex and unsupervised datasets [25, 26]. There are four primary learning methods in supervised ML (SML), unsupervised, semisupervised, and reinforcement learning, each suited for different tasks. Classic ML methods used in medicine include linear regression, logistic regression, and decision trees (DTs)/random forests (RFs) [27, 28]. Convolutional neural networks (CNNs), recurrent neural networks (RNNs), autoencoders (AE), generative adversarial networks (GANs), deep belief networks (DBNs), and hybrid architectures (HAs) are considered DL models [29]. Currently, CNNs are among the most frequently employed DL architectures [30]. These models are used for feature extraction tasks that preserve spatial data configuration and ensure translation invariance, making them commonly used in image analysis [31].

In recent years, AI has been integrated into the field of medicine to enhance patient care through faster and more precise processes, holding great promise in revolutionizing healthcare by enhancing disease detection, diagnosis [32, 33], medical imaging [34, 35], treatment planning and PM [36, 37], drug discovery [38, 39], predictive analytics, and risk assessment [40, 41]. Owing to the complex and ever-changing nature of sepsis, there is an increasing demand for personalized sepsis management [42]. A shortage of particular indicators has hindered attempts to classify patients with septic diseases based on their characteristics up until this point [42], but we have a lot of optimism which PM will enhance results for sepsis patients [43]. PM is a medical concept that combines molecular information gathered from the biological system to clinical data from every individual [44] to customize choices, methods, efforts, and treatments according to their anticipated reaction or disease risk [43]. Put differently, PM aids in the creation of a more precise molecular taxonomy of diseases, improving the identification and treatment of illnesses, and allowing for customized management of illnesses based on the unique traits of every individual [2].

Rather than replacing healthcare professionals, AI tools are expected to streamline administrative tasks, improve clinical documentation, support patient outreach, enhance medical imaging analysis, automate medical devices, and facilitate patient monitoring, ultimately optimizing healthcare delivery [45]. Also, ChatGPT, an advanced AI language model by OpenAI, generates human-like responses and can assist in diagnosis [46], education [47], and patient management [48]. A study demonstrated the potential of human–AI collaboration to improve decision-making accuracy [49]. Also, another study emphasized the need for AI to support intermediate decision-making stages by presenting SepsisLab, a system that predicts sepsis progression, visualizes uncertainty, and suggests additional tests. Evaluations with clinicians indicated that SepsisLab enhances human-AI collaboration, improving the potential for AI-assisted diagnosis in sepsis and other critical medical decisions [50].

## 3. AI Models in Early Prediction and Diagnosis of Sepsis

### 3.1. Challenges in Sepsis Detection and the Role of Biomarkers

AI is starting to be used in the diagnosis, prediction, and treatment of several life-threatening conditions, including sepsis, as its application in medicine continues to grow and now includes the ICU [51, 52]. Distinguishing sepsis from disease states (like inflammation) that share comparable clinical indications (like altered vital signs), symptoms (like fever), and molecular manifestations (like dysregulated host response) is a significant obstacle to early detection [53, 54]. In the recent pandemic of COVID-19, clinicians faced this issue significantly [55]. The systemic aspect of sepsis has led to the proposal of biomarkers, which are biological and molecular correlates, to improve the detection and diagnosis of the condition [56]. So far, despite significant attempts to develop appropriate biomarkers, no single biomarker or combination of biomarkers has been found to be widely acceptable for the diagnosis and treatment of sepsis. This is mostly because of the biomarkers' low sensitivity and specificity [57, 58].

Laboratory-tested ML algorithms have garnered attention in recent years. In SML, algorithms are trained on labeled data. These algorithms automate categorization and regression, which predict categorical and continuous outcomes, respectively [19]. A task-driven approach to supervised learning employs algorithms such as logistic regression, RF, DTs, and naïve Bayes. Clinical features help these algorithms predict disease development, but they need labeled data to train. Logistic regression and DTs are simple and reliable [59].

### 3.2. ML Approaches for Sepsis Prediction

Anjana et al. [60] conducted a comprehensive review examining the effectiveness of ML algorithms in improving sepsis diagnosis and prediction. Different classifiers have different features for detecting sepsis on balanced datasets; the RF classifier shows balanced sensitivity (0.957) and specificity (0.956) and exhibits exceptional accuracy (0.956). Its low sensitivity (0.010) and high accuracy (0.982) on imbalanced datasets suggest difficulties in identifying the minority class. On balanced datasets, XGBoost shows balanced recall (0.719) and precision (0.857), while sensitivity (0.719) and specificity (0.925) are relatively high. However, it maintains a high level of accuracy (0.982), but it faces difficulties with sensitivity (0.006) on datasets that are imbalanced. The Naive Bayes classifier excels in accuracy but fails with sensitivity when evaluated on balanced datasets (0.134). This is in contrast to its strong performance on imbalanced datasets (0.929). Despite working with balanced datasets, logistic regression obtains a reasonable accuracy of 0.753 with balanced precision and recall. However, when working with imbalanced datasets, it maintains a high accuracy of 0.982 but displays an extremely low sensitivity of 0.001. In terms of minority class correct classification using K-means and hierarchical clustering, both dataset types show minimal sensitivity to highlight conditions. On balanced datasets, isolation forest can classify both classes with moderate sensitivity (0.191) and high specificity (0.945). Despite keeping a relatively high specificity (0.903), it encounters difficulties with sensitivity (0.188) on imbalanced datasets. RF's high sensitivity makes it ideal for imbalanced datasets, in which XGBoost maintains accuracy while balancing recall and precision. The strengths of both RF and XGBoost could be leveraged in an ensemble model to improve the accuracy and reliability of classifiers [60]. They stressed on the importance of optimizing hyperparameters through methods such as Bayesian optimization, underscoring the importance of fine-tuning models to achieve the best balance between sensitivity and specificity.

As demonstrated in Table 1, several AI methods for sepsis prediction have recently been investigated. Adult patients referred to the ICU who did not meet the criteria for sepsis at the time of admission were retrospectively analyzed by Scherpf et al. Three, six, and 12 h before the onset of sepsis, they examined the sequence length provided to the ML algorithms in order to assess their prediction performance. Their neural network gets an AUROC of 0.81 for a prediction made 3 h before sepsis starts, while the InSight algorithm at this time attains an AUROC of 0.72. According to their findings, a recurrent neural network outperformed InSight in terms of prediction accuracy. They demonstrate that the look-back duration has a substantial impact on classifier performance [65]. Another study confirmed that InSight, an algorithm for predicting sepsis, severe sepsis, and septic shock based on ML, could accurately detect and forecast these conditions in a mixed-ward population. This population included patients from the floor units, ED, and ICU, and it only required six vital signs. This ML technique, namely “InSight scores,” incorporated information from six clinical vital signs: temperature, pulse rate, peripheral capillary oxygen saturation, respiration rate, and systolic and diastolic blood pressure. An ML algorithm called gradient tree boosting was built. The DT was used to compute the patient's risk score. After that, they contrasted the “InSight” forecasts for three popular scoring systems: SIRS, MEWS, and SOFA by examining trends and correlations between vital sign measurements, “InSight” was able to provide predictive capabilities prior to the start of sepsis and outperformed MEWS, SIRS, and SOFA in screening for severe sepsis, septic shock, and sepsis. With an AUROC curve of 0.92 for mild sepsis and 0.87 for severe sepsis, InSight is a very effective diagnostic tool. With an AUROC of 0.96 and an AUROC of 0.85, respectively, InSight can forecast the onset of septic shock and severe sepsis 4 h prior to the event [84].

ML assists not only in prediction but also in prompt management and treatment. Patients with sepsis have the best chance of recovery if they are identified and treated quickly [85]. Since effective treatment is time-dependent, positive outcomes are strongly correlated with efficient management in emergency departments (EDs) and wards. The transition of patients from the ED or the ward to the ICU is not always a successful solution [86]. A recent meta-analysis of 28 papers that examined the use of ML to predict sepsis found that the diagnostic test accuracy was 0.68–0.99 in the ICU, 0.96–0.98 in the hospital, and 0.87–0.97 in the ED when assessed using AUROC. ML algorithms can discriminate well and reliably predict when sepsis will start, according to this meta-analysis. One important aspect of model performance is the adoption of variables having a strong reputation for their clinical value in sepsis [87].

ML models are not limited to existing diagnostic techniques; these models are also being used to improve new diagnostic approaches [88]. Rawson et al. created an SML system to diagnose infection when a patient arrives at the hospital. The SML algorithm was trained using microbiological data records and blood test parameters, such as WBC and CRP. A binary classifying algorithm called a support vector machine (SVM) was subsequently developed. The diagnostic ability of the SVM algorithm was evaluated by training and testing it on several individual patient profiles that included biochemical and CBC information. A CDSS incorporates ML modules specifically developed to assist in the selection of antimicrobials and optimize dosage. Additionally, a patient interaction module has been implemented. Subsequently, the researchers examined individuals who were admitted to the hospital for a period of 6 months and systematically entered their data into the SML algorithm. The AUROC of 0.84 with a 95% confidence interval (CI) ranges from 0.76 to 0.91 [89].

Horng et al. (2017) conducted a retrospective study at a tertiary hospital to improve sepsis detection at ED triage using ML. They trained four ML models on a dataset of over 230,000 patient visits. Patient demographics, vital signs, chief complaint, and nurse assessment (also known as the triage note) were among the pieces of data used to initiate a procedure. Using progressively larger data subsets, they subsequently trained ML systems to forecast the infection as stated by the International Classification of Diseases, Ninth Revision, Clinical Modification (ICD-9-CM). The models included a vitals-only model, a chief complaints model, a bag-of-words model, and a topic model. The results showed that adding free text data significantly improved model performance, with the AUROC rising from 0.67 for the vitals-only model to 0.83 for the chief complaints model, and reaching 0.86 and 0.85 for the bag-of-words and topics models, respectively [61, 90].

ML algorithms for early sepsis detection are categorized as left-aligned and right-aligned models [87]. Left-aligned models predict sepsis based on a fixed time point, such as admission or preoperative status [91–93], while right-aligned models provide real-time or continuous predictions, enabling timely therapeutic interventions like drug administration [87].

### 3.3. Real-World Applications and Performance of Sepsis Prediction Models

A real-world Integration of a sepsis DL technology into routine clinical care has been conducted by Sendak et al. [94]. The study aimed to integrate Sepsis Watch, a DL-based sepsis detection system, into routine clinical care at Duke University Hospital. In 2016, a multidisciplinary team designed a workflow to use the system within the ED, utilizing patient data to trigger real-time alerts for sepsis. The system was built to update sepsis risk scores every hour and identify patients meeting sepsis criteria every 5 min. The ML model incorporated both static (e.g., demographics) and dynamic (e.g., vital signs, medication) features. During the 6-month pilot, Sepsis Watch maintained a 99.34% system uptime. On average, 14 patients met sepsis criteria daily, and 7 were at high risk. The integration led to improvements in sepsis bundle compliance, with 62.16% of patients meeting 3-h antibiotic compliance and 89.83% meeting blood culture collection compliance before the system's implementation. While still under evaluation, Sepsis Watch significantly improved clinical decision-making, helping streamline sepsis management in the ED [94]. In a recent study by Valan et al. [95], the generalizability of the Sepsis Watch ML model was evaluated in detecting sepsis in a new community health system, Summa Health, by validating its performance across four EDs. The dataset included 205,005 encounters from 101,584 unique patients, with an average age of 50 years. The Sepsis Watch model demonstrated strong performance with AUROC ranging from 0.906 to 0.960 across the four sites. The model maintained robust accuracy even when stratified by patient ethnicity, showing minimal variation in performance across different demographics. The study also explored the balance between alert fatigue and clinical benefit, showing that varying the model's prediction threshold allowed for optimization of recall and precision. The study highlights Sepsis Watch's strong generalizability, suggesting its potential for implementation in diverse healthcare settings with minimal adaptation, although further prospective studies are needed to evaluate its impact on patient outcomes [95].

In another study, researchers have developed DL-based prediction model. Boussina et al. [96] reported a significant advancement in the application of DL for sepsis prediction through their quasiexperimental study deploying the COMPOSER model in two EDs. Their real-time algorithm integration was associated with a 1.9% absolute reduction in in-hospital sepsis mortality and a 5.0% absolute increase in sepsis bundle compliance, outcomes that translate to meaningful clinical benefits including reduced organ dysfunction as measured by 72-h SOFA score changes. Notably, this study demonstrated improved timeliness of antibiotic administration linked to nursing staff engagement with the predictive alerts, highlighting an effective human–AI interface that mitigates alarm fatigue by employing conformal prediction to reduce false positives. Unlike prior sepsis prediction models with poor positive predictive values (PPVs) and limited bedside validation, COMPOSER's real-world implementation and robust performance underscore the potential of DL algorithms to positively influence patient-centered outcomes in acute care settings [96].

## 4. AI-Enhanced Sepsis Management Strategies

### 4.1. CDSSs and Implementation of AI

**Figure 1:**
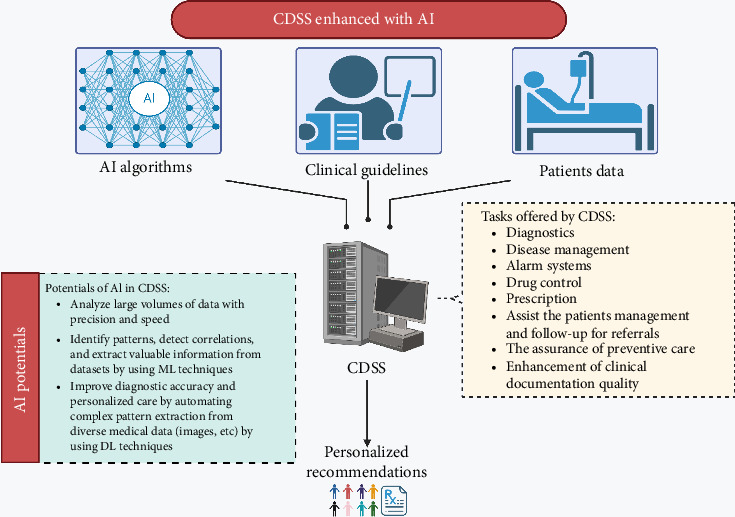
Integration of artificial intelligence (AI) algorithms into clinical decision support systems (CDSS). AI-integrated systems can analyze large data volumes, enhance diagnostics, and provide personalized recommendations for disease management, drug control, and patient follow-up.

By supplementing medical choices with patient information, specialized clinical knowledge, and other health-related data, a CDSS seeks to improve healthcare delivery (Figure 1) [97]. CDSSs are commonly categorized as either knowledge-based or non–knowledge-based. Knowledge-based systems utilize IF-THEN statements, which are generated to establish rules. [98]. Rules can be formulated based on literature, practice, or patient-directed evidence [99]. The decision-making process in non–knowledge-based CDSS still relies on a data source, but instead of being programmed to follow expert medical knowledge, it employs AI, ML, or statistical pattern recognition [98]. The range of tasks offered by CDSS is various, including diagnostics, disease management, alarm systems, drug control, prescription (Rx), and numerous more capabilities [100] and also transformed the potentials of CDSS, allowing it to efficiently analyze and understand large volumes of healthcare data with unparalleled precision and speed [101]. For instance, research has demonstrated that CDSS can effectively enhance compliance with clinical guidelines [102]. Additionally, it can assist the patient management involved in research or treatment protocols [103], the follow-up for referrals, the monitoring and placement of orders, the assurance of preventative care [104], and the enhancement of clinical documentation quality [105]. In addition, Chen et al. highlighted the flexibility and extensive usefulness of CDSS, showcasing its effectiveness in a wide range of healthcare environments, from primary care clinics to ICUs [106].

The integration of AI and ML has significantly enhanced CDSS, improving efficiency and precision in healthcare [101, 107]. Studies highlight ChatGPT's adaptability in CDSS, expanding its role in decision-making and patient management [108]. ML algorithms, including DTs and DL models, analyze complex medical data, detect patterns, and provide personalized treatment recommendations [109]. DL further enhances diagnosis accuracy and supports medical imaging, genomics, and natural language processing (NLP) [110]. By leveraging diverse data sources, AI-driven CDSS facilitates early disease detection [110], optimized treatment planning, and PM, ultimately improving patient outcomes and therapy effectiveness [111].

Although AI technologies have great potential to revolutionize CDSS and enhance healthcare delivery, their incorporation into clinical practice is accompanied by significant obstacles that need to be resolved in order to guarantee its efficacy, safety, and ethical application. The hurdles encompass technical restrictions, regulatory restraints, ethical issues, and organizational barriers that affect the implementation and expansion of AI-driven CDSS [112]. The ethical challenges in PM necessitate continuous attention, including the responsible use of AI-generated insights, data ownership, and consent [113]. Furthermore, Wan et al. draw attention to the CDSS's comparatively poor performance in managing rare cases and sporadic false positives in particular high-risk circumstances [114].

### 4.2. AI Applications in Optimizing Antibiotic Therapy

ML has been utilized in many ways to address AMR, including antibiotic resistance (ABR) [15]. One example is the use of sequence-based AI in the study of AMR [115–117]. AI has been utilized to create novel antibiotics and produce a synergistic effect by combining multiple medications [118, 119]. ML techniques anticipate drug-resistant microorganisms by analyzing antimicrobial use and resistance data. This can inform healthcare practitioners about drug selection and usage [120]. ML is also used to optimize antibiotic use in healthcare by determining the best treatment combination for an infection or predicting which patients will acquire a resistant infection [14, 16].

The development of ABR is primarily driven by inherent genetic alterations and transferable antibiotic resistance genes (ARGs). AI technologies and approaches have the ability to enhance the detection of ARGs and identify antibiotic targets and compounds that can act as antibiotics by killing or inhibiting the growth of bacteria. Popular techniques for identifying and annotating ARGs include SVM, hidden Markov models (HMM), and neural networks. The feature selection component of RF and eXtreme Gradient Boosting (XGBoost) algorithms are effective in recognizing possible ARGs [121]. An SVM model achieved the highest performance in distinguishing between pathogenic and nonpathogenic bacterial proteins [122]. One of the most well-known DL-based ARG detection systems is DeepARG, and a collection of ANN models, DeepARG-LS, and DeepARG-SS identifies ARGs from short reads and assembled sequences [123].

One significant application of AI is in the development of CDSS. These systems can predict the likely AMR of bacteria even before definitive culture results are available. For instance, ML models have shown promising results in predicting susceptibility at various stages of the bacteriological process, such as during specimen sampling, direct examination, and culture growth stages [14, 124]. A notable study applied ML to predict AMR using a database of over 44,000 patient cases. In this study, Gao et al. developed ML models to predict AMR in *Acinetobacter baumannii* using whole-genome sequencing (WGS) data. The researchers utilized three algorithms—RF, SVM, and XGBoost—to predict the minimum inhibitory concentrations (MICs) for 13 antimicrobial agents, based on k-mer features extracted from the genomic sequences of 339 isolates. The models were trained on an 80% training set and tested on a 20% testing set. RF outperformed the other models, achieving high essential agreement (90.90%) and category agreement (95.29%), with minimal very major error rates (0.0% to 5.71%). The study demonstrated that this ML approach could predict MICs in just 10 min, offering a rapid alternative to traditional antimicrobial susceptibility testing, which typically takes 36 h, and showing great promise for clinical applications in identifying AMR in *A. baumannii* [125].

In another significant study, XGBoost predicted futuristic AMR prevalence across 119 English hospital trusts using historical AMR data (2016–2022) and antibiotic usage records for 22 pathogen–antibiotic combinations, including E. coli, Klebsiella spp., methicillin-susceptible coagulase-positive Staphylococcus species (MSSA), and *P. aeruginosa*. Results showed that XGBoost outperformed baseline methods, reducing the mean absolute error (MAE) by up to 2% (e.g., from 6% to 4% for *P. aeruginosa* ceftazidime resistance) and achieving 30% better predictions in trusts with > 10% annual AMR fluctuations. Key predictive features included historical resistance for the same pathogen–antibiotic combination, cross-pathogen resistance patterns, and antibiotic usage trends, such as rising amoxicillin/clavulanic acid use (24% to 32% of bed-days) and declining trimethoprim use (8% to 3%). While 84% of trust–pathogen–antibiotic combinations exhibited < 5% annual resistance variability, XGBoost's ability to model complex interactions enabled accurate forecasting, particularly for high-resistance scenarios (e.g., 43% median resistance for E. coli–amoxicillin/clavulanic acid), supporting targeted AMR surveillance and stewardship interventions [124].

Chronobiology affects organ function, particularly the immune system [126]. On the other side, many parasites, viruses, and bacteria have circadian rhythms [127]. Yotam Kolben et al. proposed a second-generation AI chronobiology-based strategy to prevent and maybe overcome resistance. An algorithm-controlled regimen is being developed to improve the long-term efficiency of antimicrobial medicines by introducing diversity in dose and medication administration periods. The method ensures sustainability and better results [128].

### 4.3. Real-Time Monitoring and Alert Systems for Sepsis Progression

**Figure 2:**
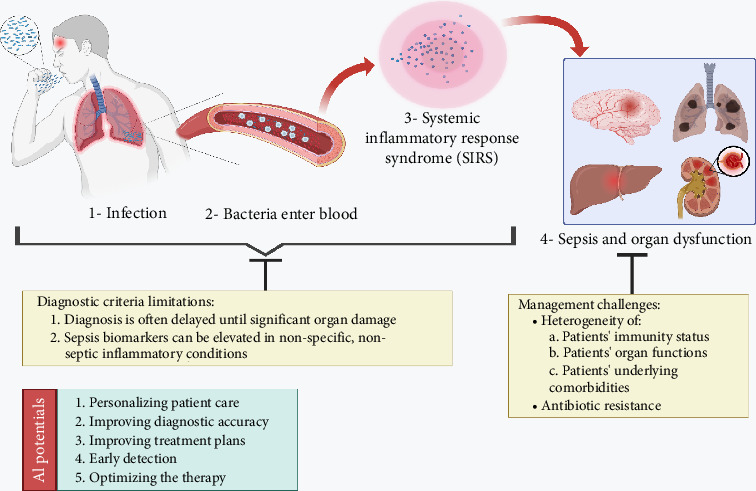
The potential of artificial intelligence (AI) in progression of sepsis, from bacterial entry to the development of systemic inflammatory response syndrome (SIRS) and subsequent organ dysfunction. The diagnostic limitations of traditional criteria like SOFA and SIRS might lead to late diagnosis of sepsis only after significant organ damage. Artificial intelligence (AI) showed promise in enhancing sepsis management, including personalized care, early detection, improved diagnostic accuracy, and optimization of therapy.

Consistently following early treatment interventions, especially administering antibiotics effectively, is linked to better outcomes and reduced cost [129–133]. To improve sepsis outcomes, the CMS instituted a sepsis management bundle as a fundamental quality indicator. Gathering blood cultures, serum lactate, starting intravenous fluids, and broad-spectrum antibiotics are all part of this bundle of timely procedures [134]. Information technology has the potential to enhance early sepsis care by providing physicians with real-time monitoring and automated alarms if they are in danger of bundle nonadherence (Figure 2) [135].

A real-time sepsis care monitoring and paging alert system substantially increased the delivery of 3-h adherent care and the number of orders for suspected sepsis patients who were at risk of receiving 3-h bundle nonadherent care. Nevertheless, even though patients with a clear clinical suspicion of sepsis were included in the study, it was shown that early withdrawal of antibiotics and negative culture results were frequent. This emphasizes the difficulties in accurately identifying the right patients for the sepsis bundle application [135].

The sepsis alert implemented at Barnes–Jewish Hospital has shown a significant improvement in the timely implementation of therapeutic and diagnostic measures for non-ICU patients who are susceptible to sepsis [136]. The sensitivity of automated sepsis alerts in the ED can be adjusted to a high level. The process outcomes indicate a moderate level of advantage. Nevertheless, there is no single measure that has consistently shown improvement, and high-quality studies have not yet demonstrated an advantage in reducing mortality [137]. During a quality improvement initiative at a single medical center, the use of an electronic health record-based sepsis early warning system, along with pharmacist notification through the electronic health record, resulted in a shorter time for administering antibiotics. This improvement was achieved without any negative or potentially harmful clinical interventions [138]. Automated sepsis alarms generated from electronic health data have the potential to enhance treatment processes; however, they often exhibit low PPV and do not result in improvements in mortality rates or length of hospital stays [139].

There is a lack of sufficient information evaluating the effect of prehospital sepsis alarm systems on the identification and management of sepsis [140]. Retrospective analysis indicates that the prehospital sepsis alert strategy cannot reduce the time required to complete the CMS sepsis management bundle. Future research should focus on the ability of Emergency Medical Services (EMS) clinicians to identify sepsis and evaluate the effectiveness of sepsis therapies initiated by EMS clinicians, such as administering antibiotics [134].

As demonstrated in Table 2, another significant consideration is ethical considerations, particularly around issues of fairness, privacy, and accountability. One key concern is ensuring that AI systems are trained on diverse, representative data to avoid bias that could lead to unequal treatment across different patient groups. There are also questions about patient consent and the transparency of AI decision-making processes, as these systems often function as “black boxes,” making it difficult to understand how they arrive at conclusions. Ethical guidelines are essential to balance the benefits of AI in improving healthcare with the need to protect patient rights and ensure equitable care. On January 6, 2025, the FDA released draft guidelines for using AI in the drug and biological product lifecycle. The guidance provides a seven-step process to ensure the credibility of AI models, focusing on risk assessment and establishing pre- and postcredibility evaluations. It emphasizes the importance of understanding AI model risks and encourages early discussions between manufacturers and the FDA about credibility assessment plans. The guidelines aim to foster collaboration between manufacturers and the FDA, offering a flexible framework to address key questions, rather than imposing rigid rules, as AI's role in drug development continues to evolve [160].

## 5. Conclusion

Sepsis remains a leading cause of morbidity and mortality in critically ill patients, necessitating timely and accurate diagnosis to improve outcomes. Traditional diagnostic methods, including SOFA, SIRS, and qSOFA, often fail to detect sepsis at an early stage, limiting opportunities for timely intervention. AI has emerged as a transformative tool in sepsis management, leveraging ML, DL, and NLP to enhance early detection, optimize treatment strategies, and enable precision medicine. AI-driven CDSSs have shown immense potential in improving diagnostic accuracy, optimizing antimicrobial therapy, and enabling real-time patient monitoring. By integrating AI with EHRs, predictive models can analyze vast amounts of patient data, identify patterns indicative of sepsis progression, and facilitate early intervention.

Various AI models have been explored for sepsis prediction and management, each with unique capabilities. Supervised learning models, such as logistic regression, DTs, and RFs, have been widely used for classification and risk prediction. More advanced techniques, including gradient boosting machines (e.g., XGBoost), RNNs, and CNNs, have demonstrated higher predictive accuracy by analyzing complex, high-dimensional data. Ensemble models, which combine multiple algorithms, further enhance robustness and reliability. Additionally, unsupervised learning methods, such as clustering algorithms (e.g., K-means), help identify hidden patterns and patient subgroups, refining risk stratification and personalized treatment approaches. The integration of reinforcement learning in adaptive treatment planning represents a promising direction for future AI applications in ICUs.

Despite its promise, AI implementation in sepsis management faces significant challenges, including data heterogeneity, algorithmic bias, regulatory constraints, and ethical considerations. The variability in healthcare settings, differences in patient populations, and the need for transparent, explainable AI models highlight the necessity for robust multicenter validation studies, standardized regulatory frameworks, and interdisciplinary collaborations. Moving forward, continued research, investment in AI-driven healthcare infrastructure, and integration of explainable AI models into clinical practice are essential. Achieving its full potential requires ethical AI deployment, regulatory oversight, and clinician-AI collaboration to ensure safe, equitable, and effective implementation.

## Figures and Tables

**Table 1:** Predictive and diagnostic potential of artificial models.

Type of study	Year	Utility	Section	Patients	Model	Number of variants	AUROC	Sensitivity	Specificity	Accuracy	Ref
Retrospective	2017	Predictive	Emergency department (ED)		Support vector machine (SVR)		0.86	0.81	0.75		[61]

Retrospective	2019	Diagnostic	ED		Gradient-boosted tree model	13	0.93 to 0.97				[62]

Retrospective	2018	Predictive	Hospital-wide	Patients diagnosed with pneumonia				99.74% ± 0.13%		98.63 ± 0.17%	[63]

Retrospective	2019	Predictive	Hospital-wide	Non-ICU admissions			0.88	0.26	0.98		[64]

Retrospective	2019	Predictive	ICU	ICU admissions	Recurrent neural network	10	0.81		0.47		[65]

Retrospective	2017	Predictive	ICU	ICU admissions	Support vector machine (SVM)	2	0.8			61%	[66]

Retrospective	2019	Predictive	ICU	Critically ill patients	Random forest	7	0.79	0.80			[67]

Retrospective	2018	Predictive	ICU	ICU admissions	Weibull–Cox proportional hazards model	48	0.85	0.85			[68]

Retrospective	2016	Predictive	ICU	ICU admissions	InSight	9	0.92	0.90	0.81		[69]

Retrospective	2015	Predictive	ICU	ICU admissions	Logistic regression		0.877	0.603	0.932		[70]

Retrospective	2015	Predictive	ICU	ICU admissions	SVM		0.871	0.642	0.936		[70]

Retrospective	2015	Predictive	ICU	ICU admissions	Logistic model tree;		0.882	0.609	0.933		[70]

Retrospective	2022	Differential diagnostic	Hospital-wide		Random forest			0.95	0.80	0.949	[71]

Retrospective	2020	Predictive	ICU		LSTM (ClinicalBERT)	10	0.84	0.67	0.85	—	[72]

Retrospective	2021	Predictive and diagnostic	ICU	ICU admission with an ICD-10 code for sepsis, severe sepsis, or sepsis shock	Logistic regression	14	0.94 (12 h before the onset)	0.87	0.87		[73]

Retrospective	2024	Predictive	NICU	Neonates admitted in NICU	Random forest	—	—	—	0.994	0.98	[74]

Retrospective	2024	Predictive of mortality	ICU		Random forest		0.94				[75]

Retrospective	2020	Predictive	ICU		Lasso regression		0.89 30.64 h		0.67		[76]

Retrospective	2024	Predictive of mortality	ICU	ICU admission	XGBoost model	14	0.806 (predicting 28-day mortality)				[77]

RCT	2023	Predictive	ICU	Patients with acute pancreatitis	Gradient boosting decision tree (GBDT)	13	0.985	0.996	0.992	0.994	[78]

Retrospective	2022	Prognostic	ICU	Patients with sepsis	eXtreme Gradient Boosting (XGBoost) model	5	0.884			0.895	[79]

Retrospective	2021	Prognostic (predicting septic shock)	ICU	Patients with sepsis	Random forest	15	0.9483	0.839	0.881		[80]

Retrospective	2021	Prognostic (prediction of delayed septic shock)	ED	Patients with sepsis	Artificial intelligence sepsis expert	40	(> 0.8) at 8 and 12 h	0.85	0.678		[81]

Retrospective	2020	Predictive	ICU		Bidirectional gated recurrent unit	36	0.97 in 6 h			0.998	[82]

RCT	2022	Predictive for postoperative healthcare-associated infection (HAI)	Hospital-wide	Patients with radical resection of advanced digestive system tumor	Deep learning–based prediction model		0.728			0.733	[83]

**Table 2:** Challenges and solutions in the implementation of AI in healthcare.

Concerns	Explanation	Root causes	Potential solutions	Barriers to implementation
Algorithmic bias	AI algorithms must be globally inclusive and tested on diverse populations to ensure fair and effective clinical outcomes [141].	This occurs when algorithms originate from data from majority groups, so they exhibit poor outcomes for groups that lack adequate representation in the dataset [141].	Raise awareness of these concerns and provide physicians the ability to engage critically in the design and development of systems [141].	

Lack of adequate data	Most studies have used single-center retrospective designs, which are insufficient for creating reliable and generalizable AI tools [142, 143].	The algorithm is trained exclusively on the database where it was initially constructed, without testing it on an external cohort [144, 145].	Use multicenter studies	Meeting privacy and regulations while gathering diverse information is challenging. Transferring a model to another institution may involve technical variations, local practices, and patient types. Access to data and computer science skills is also crucial [146].

Data shift	A difference in data distribution among learning models and real-world data contexts [147].	The evolution of day-to-day procedures, healthcare systems, and patient populations throughout time [147].	Regular performance monitoring, retraining, and data-driven testing are essential to detect issues early and maintain AI model accuracy [148, 149].	

Comparison of algorithms	The success of every study has been demonstrated through various approaches on diverse populations, each with its unique sample distribution and features [141].	Area under the receiver operating characteristic curve (AUROC) is widely used to assess prediction models but can be misleading due to its inability to account for prior probability, error distribution, and proper weighting of omission and commission errors [150, 151].	Algorithms must undergo multiple tests that are acknowledged by all for accurate and adequate assessments [141]. Tests should assess model sensitivity and specificity at clinically meaningful thresholds, along with chance ratios indicating how odds change post-test compared to pretest [146]. The test set should be tailored to the specific target population [141].	

Ununified data	Data are often fragmented across multiple systems (e.g., EHRs, pathology, prescriptions, and insurance), making integration and consolidation challenging [141].	Healthcare systems make it hard to do real-time research because data is not always provided [141].	Data formats that are unified, including the fast healthcare interoperability resources [152].	It is necessary to make sufficient investments in local analytical system in order to enable collecting data, curation, transformation, and analytics [152].

Human barriers	For healthcare professionals to make certain that this technology is able to reach patients and provide them with benefits, it will be necessary to get a deeper comprehension of the interactions that take place between humans and computers [141].	N/A	The final consumers of AI systems or healthcare workers should get regular and proper training, and human factors should be tested to make sure AI is used correctly [153].	

Explainability	AI systems have limitations in their usefulness as they cannot provide an “explanation” for their decision-making processes [154]. The recognition of faults and the detection of bias or confounding factors are only possible through a system that can be explained [146].	Understanding the mathematical principles behind models is challenging and often unattainable, making it difficult to investigate their inner workings and their decision-making processes [141].	Shapley additive explanation (SHAP) uses equal profit distribution among stakeholders to interpret gradient boosting decision tree models, particularly in machine learning models, revealing relationships between features and outcomes, particularly in complex and highly sophisticated ensemble decision trees [155]. The problem can be effectively addressed by actively engaging clinical end users and consumers at every stage of product development [146].	

Interaction between human and algorithm	During the pre-deployment stage of an AI system, it's crucial to determine if the system's outcomes will be managed by a specific team or attending physicians [146]. System alerts must balance sensitivity and alert fatigue, considering sepsis' clinical significance, high occurrence, and patient population severity [156].	A selected group's control of system results seems to alleviate alarm-related fatigue in attending physicians, but some may find this approach disruptive [146].	Alerts, along with additional suggestions or data, may be sent to physicians via email, phone, or electronic health record systems at the medical facility [94].	

Regulatory frameworks	An essential element in the process to accomplish the deployment of AI algorithms in a secure and efficient manner [146].	The rapid pace of innovation, significant risks, and the dynamic nature of machine learning models present a significant issue [141].	The FDA has addressed concerns about AI systems in healthcare, but many systems lack a regulatory framework to ensure secure implementation [157].	

Data without proper validation	Getting high-quality data that has not been properly checked is hard when making AI models [107].	Most of the data used in AI experiments to predict sepsis comes straight from hospitals' electronic health record systems and has not been checked or validated [107].	Various proposals have been proposed to address this issue, including the use of signal estimators or moving average models [158, 159].	

## Data Availability

Data sharing is not applicable to this article as no new data were created or analyzed in this study. All data used in the manuscript were sourced from published literature and publicly available databases.

## Nomenclature

AI: Artificial intelligence
ANN: Artificial neural network
ARG: Antibiotic resistance gene
AUROC: Area under the receiver operating characteristic curve
CDSS: Clinical decision support system
DL: Deep learning
ED: Emergency department
ICU: Intensive care unit
ML: Machine learning
NLP: Natural language processing
PM: Personalized medicine
SIRS: Systemic inflammatory response syndrome
SOFA: Sequential Organ Failure Assessment
SVM: Support vector machine

## References

[1] Singer M., Deutschman C. S., Seymour C. W. (2016). The Third International Consensus Definitions for Sepsis and Septic Shock (Sepsis-3). *JAMA*.

[2] Rudd K. E., Johnson S. C., Agesa K. M. (2020). Global, Regional, and National Sepsis Incidence and Mortality, 1990–2017: Analysis for the Global Burden of Disease Study. *The Lancet*.

[3] van Vught L. A., Klein Klouwenberg P. M. C., Spitoni C. (2016). Incidence, Risk Factors, and Attributable Mortality of Secondary Infections in the Intensive Care Unit After Admission for Sepsis. *JAMA*.

[4] Markwart R., Saito H., Harder T. (2020). Epidemiology and Burden of Sepsis Acquired in Hospitals and Intensive Care Units: A Systematic Review and meta-analysis. *Intensive Care Medicine*.

[5] Bone R. C., Balk R. A., Cerra F. B. (1992). Definitions for Sepsis and Organ Failure and Guidelines for the Use of Innovative Therapies in Sepsis. *Chest*.

[6] López-Izquierdo R., Brio-Ibañez P., Martín-Rodríguez F. (2020). Role of qSOFA and SOFA Scoring Systems for Predicting In-Hospital Risk of Deterioration in the Emergency Department. *International Journal of Environmental Research and Public Health*.

[7] Moeini Taba M., Eshraghi R., Zare Tutestani M., Moraveji S. A., Sehat M., Banafshe H. R. (2024). Evaluating Convalescent Plasma Therapy in Severe COVID-19: A Retrospective Cohort Study. *The Journal of Infection in Developing Countries*.

[8] Duncan C. F., Youngstein T., Kirrane M. D., Lonsdale D. O. (2021). Diagnostic Challenges in Sepsis. *Current Infectious Disease Reports*.

[9] Singer M. (2020). Biomarkers for Sepsis–Past, Present and Future. *Qatar Medical Journal*.

[10] Pant A., Mackraj I., Govender T. (2021). Advances in Sepsis Diagnosis and Management: A Paradigm Shift Towards Nanotechnology. *Journal of Biomedical Science*.

[11] Komorowski M., Green A., Tatham K. C., Seymour C., Antcliffe D. (2022). Sepsis Biomarkers and Diagnostic Tools With a Focus on Machine Learning. *EBioMedicine*.

[12] Santacroce E., D’Angerio M., Ciobanu A. L. (2024). Advances and Challenges in Sepsis Management: Modern Tools and Future Directions. *Cells*.

[13] Ranjit S., Kissoon N. (2021). Challenges and Solutions in Translating Sepsis Guidelines Into Practice in Resource-Limited Settings. *Translational Pediatrics*.

[14] Ali T., Ahmed S., Aslam M. (2023). Artificial Intelligence for Antimicrobial Resistance Prediction: Challenges and Opportunities Towards Practical Implementation. *Antibiotics*.

[15] Thakral Y., Sahay S., Mukherjee A. (2022). Strengthening Digital Monitoring of Antibiotic Resistance in Low-Resource Settings. *Journal of Global Health*.

[16] Javaid M., Haleem A., Pratap Singh R., Suman R., Rab S. (2022). Significance of Machine Learning in Healthcare: Features, Pillars and Applications. *International Journal of Intelligent Networks*.

[17] Talat A., Khan A. U. (2023). Artificial Intelligence as a Smart Approach to Develop Antimicrobial Drug Molecules: A Paradigm to Combat Drug-Resistant Infections. *Drug Discovery Today*.

[18] Steinbach D., Ahrens P. C., Schmidt M. (2024). Applying Machine Learning to Blood Count Data Predicts Sepsis With ICU Admission. *Clinical Chemistry*.

[19] Agnello L., Vidali M., Padoan A. (2024). Machine Learning Algorithms in Sepsis. *Clinica Chimica Acta*.

[20] Persson I., Macura A., Becedas D., Sjövall F. (2024). Early Prediction of Sepsis in Intensive Care Patients Using the Machine Learning Algorithm NAVOY® Sepsis, a Prospective Randomized Clinical Validation Study. *Journal of Critical Care*.

[21] (2022). *Artificial Intelligence Technology*.

[22] Fathi M., Eshraghi R., Behzad S. (2024). Potential Strength and Weakness of Artificial Intelligence Integration in Emergency Radiology: A Review of Diagnostic Utilizations and Applications in Patient Care Optimization. *Emergency Radiology*.

[23] Fathi M., Vakili K., Hajibeygi R. (2025). Cultivating Diagnostic Clarity: The Importance of Reporting Artificial Intelligence Confidence Levels in Radiologic Diagnoses. *Clinical Imaging*.

[24] Hajikarimloo B., Mohammadzadeh I., Nazari M. A. (2025). Prediction of Facial Nerve Outcomes After Surgery for Vestibular Schwannoma Using Machine Learning-Based Models: A Systematic Review and Meta-Analysis. *Neurosurgical Review*.

[25] Alaskar H., Saba T. Machine Learning and Deep Learning: A Comparative Review.

[26] Sharifi G., Hajibeygi R., Zamani S. A. M. (2024). Diagnostic Performance of Neural Network Algorithms in Skull Fracture Detection on CT Scans: A Systematic Review and Meta-Analysis. *Emergency Radiology*.

[27] Choi R. Y., Coyner A. S., Kalpathy-Cramer J., Chiang M. F., Campbell J. P. (2020). Introduction to Machine Learning, Neural Networks, and Deep Learning. *Translational Vision Science & Technology*.

[28] Dooraki S. K., Maharat Z., Khalaji A., Pazouki L., Dooraki S. (2024). Early Detection of Celiac Disease Through Its Common Symptoms Using Machine Learning Algorithms. *Journal of Clinical Images and Medical Case Reports*.

[29] Piccialli F., Somma V. D., Giampaolo F., Cuomo S., Fortino G. (2021). A Survey on Deep Learning in Medicine: Why, How and When?. *Information Fusion*.

[30] Nogales A., Garcia-Tejedor A. J., Monge D., Vara J. S., Anton C. (2021). A Survey of Deep Learning Models in Medical Therapeutic Areas. *Artificial Intelligence in Medicine*.

[31] Rawat W., Wang Z. (2017). Deep Convolutional Neural Networks for Image Classification: a Comprehensive Review. *Neural Computation*.

[32] Noseworthy P. A., Attia Z. I., Behnken E. M. (2022). Artificial Intelligence-Guided Screening for Atrial Fibrillation Using Electrocardiogram During Sinus Rhythm: A Prospective Non-Randomised Interventional Trial. *The Lancet*.

[33] Upton R., Mumith A., Beqiri A. (2022). Automated Echocardiographic Detection of Severe Coronary Artery Disease Using Artificial Intelligence. *Journal of the American College of Cardiology: Cardiovascular Imaging*.

[34] Nam J. G., Hwang E. J., Kim J. (2023). AI Improves Nodule Detection on Chest Radiographs in a Health Screening Population: A Randomized Controlled Trial. *Radiology*.

[35] Zhang R., Wei Y., Shi F. (2022). The Diagnostic and Prognostic Value of Radiomics and Deep Learning Technologies for Patients With Solid Pulmonary Nodules in Chest CT Images. *BMC Cancer*.

[36] Auloge P., Cazzato R. L., Ramamurthy N. (2020). Augmented Reality and Artificial Intelligence-Based Navigation During Percutaneous Vertebroplasty: A Pilot Randomised Clinical Trial. *European Spine Journal*.

[37] Joshi S., Shamanna P., Dharmalingam M. (2023). Digital Twin Enabled Personalized Nutrition Improves Metabolic Dysfunction-Associated Fatty Liver Disease in Type 2 Diabetes: Results of a 1-Year Randomized Controlled Study. *Endocrine Practice*.

[38] Kennedy K., Cal R., Casey R. (2020). The Anti‐Ageing Effects of a Natural Peptide Discovered by Artificial Intelligence. *International Journal of Cosmetic Science*.

[39] Matin H., Taghian F., Chitsaz A. (2022). Artificial Intelligence Analysis to Explore Synchronize Exercise, Cobalamin, and Magnesium as New Actors to Therapeutic of Migraine Symptoms: a Randomized, Placebo-Controlled Trial. *Neurological Sciences*.

[40] Brennan M., Puri S., Ozrazgat-Baslanti T. (2019). Comparing Clinical Judgment With the Mysurgeryrisk Algorithm for Preoperative Risk Assessment: A Pilot Usability Study. *Surgery*.

[41] Sideris K., Weir C. R., Schmalfuss C. (2024). Artificial Intelligence Predictive Analytics in Heart Failure: Results of the Pilot Phase of a Pragmatic Randomized Clinical Trial. *Journal of the American Medical Informatics Association*.

[42] Christaki E., Giamarellos-Bourboulis E. J. (2014). The Beginning of Personalized Medicine in Sepsis: Small Steps to a Bright Future. *Clinical Genetics*.

[43] Watkins R. R., Bonomo R. A., Rello J. (2022). Managing Sepsis in the Era of Precision Medicine: Challenges and Opportunities. *Expert Review of Anti-infective Therapy*.

[44] Ibarz M., Haas L. E. M., Ceccato A., Artigas A. (2024). The Critically Ill Older Patient With Sepsis: A Narrative Review. *Annals of Intensive Care*.

[45] Bohr A., Memarzadeh K. (2020). The Rise of Artificial Intelligence in Healthcare Applications. *Artificial Intelligence in healthcare*.

[46] Madadi Y., Delsoz M., Lao P. A. (2023). Chatgpt Assisting Diagnosis of Neuro-Ophthalmology Diseases Based on Case Reports. *medRxiv*.

[47] Campbell D. J., Estephan L. E., Mastrolonardo E. V., Amin D. R., Huntley C. T., Boon M. S. (2023). Evaluating ChatGPT Responses on Obstructive Sleep Apnea for Patient Education. *Journal of Clinical Sleep Medicine*.

[48] Allahqoli L., Ghiasvand M. M., Mazidimoradi A., Salehiniya H., Alkatout I. (2023). Diagnostic and Management Performance of ChatGPT in Obstetrics and Gynecology. *Gynecologic and Obstetric Investigation*.

[49] A Human-AI Collaborative Approach for Clinical Decision Making on Rehabilitation Assessment.

[50] Rethinking Human-AI Collaboration in Complex Medical Decision Making: A Case Study in Sepsis Diagnosis.

[51] Topol E. J. (2019). High-Performance Medicine: The Convergence of Human and Artificial Intelligence. *Nature Medicine*.

[52] De Corte T., Van Hoecke S., De Waele J. (2022). Artificial Intelligence in Infection Management in the ICU. *Critical Care*.

[53] Lever A., Mackenzie I. (2007). Sepsis: Definition, Epidemiology, and Diagnosis. *BMJ*.

[54] Al Jalbout N., Troncoso R., Evans J. D., Rothman R. E., Hinson J. S. (2019). Biomarkers and Molecular Diagnostics for Early Detection and Targeted Management of Sepsis and Septic Shock in the Emergency Department. *Journal Applied Medicine*.

[55] Eshraghi R., Bahrami A., Karimi H. M., Nasr Azadani M. J. N. (2024). The Ongoing Battle: Unpacking the Characteristics of a New Dominant COVID-19 Variant. *Pathogens and Global Health*.

[56] Hotchkiss R. S., Moldawer L. L., Opal S. M., Reinhart K., Turnbull I. R., Vincent J. L. (2016). Sepsis and Septic Shock. *Nature Reviews Disease Primers*.

[57] Parlato M., Philippart F., Rouquette A. (2018). Circulating Biomarkers May be Unable to Detect Infection at the Early Phase of Sepsis in ICU Patients: The CAPTAIN Prospective Multicenter Cohort Study. *Intensive Care Medicine*.

[58] Faix J. D. (2013). Biomarkers of Sepsis. *Critical Reviews in Clinical Laboratory Sciences*.

[59] Tolles J., Meurer W. J. (2016). Logistic Regression: Relating Patient Characteristics to Outcomes. *JAMA*.

[60] Sontag D. A. (2024). Improving Sepsis Classification Performance With Artificial Intelligence Algorithms: A Comprehensive Overview of Healthcare Applications. *Journal of Critical Care*.

[61] Horng S., Sontag D. A., Halpern Y., Jernite Y., Shapiro N. I., Nathanson L. A. (2017). Creating an Automated Trigger for Sepsis Clinical Decision Support at Emergency Department Triage Using Machine Learning. *PLoS One*.

[62] Delahanty R. J., Alvarez J., Flynn L. M., Sherwin R. L., Jones S. S. (2019). Development and Evaluation of a Machine Learning Model for the Early Identification of Patients at Risk for Sepsis. *Annals of Emergency Medicine*.

[63] Tansakul V., Li X., Koszalinski R., Paiva W., Khojandi A. (2018). Prediction of Sepsis and In-Hospital Mortality Using Electronic Health Records. *Methods of Information in Medicine*.

[64] Giannini H. M., Ginestra J. C., Chivers C. (2019). A Machine Learning Algorithm to Predict Severe Sepsis and Septic Shock: Development, Implementation, and Impact on Clinical Practice. *Critical Care Medicine*.

[65] Scherpf M., Gräßer F., Malberg H., Zaunseder S. (2019). Predicting Sepsis With a Recurrent Neural Network Using the MIMIC III Database. *Computers in Biology and Medicine*.

[66] Shashikumar S. P., Stanley M. D., Sadiq I. (2017). Early Sepsis Detection in Critical Care Patients Using Multiscale Blood Pressure and Heart Rate Dynamics. *Journal of Electrocardiology*.

[67] van Wyk F., Khojandi A., Mohammed A., Begoli E., Davis R. L., Kamaleswaran R. (2019). A Minimal Set of Physiomarkers in Continuous High Frequency Data Streams Predict Adult Sepsis Onset Earlier. *International Journal of Medical Informatics*.

[68] Nemati S., Holder A., Razmi F., Stanley M. D., Clifford G. D., Buchman T. G. (2018). An Interpretable Machine Learning Model for Accurate Prediction of Sepsis in the ICU. *Critical Care Medicine*.

[69] Calvert J. S., Price D. A., Chettipally U. K. (2016). A Computational Approach to Early Sepsis Detection. *Computers in Biology and Medicine*.

[70] (2015). Predictive Models for Severe Sepsis in Adult ICU Patients. *Systems and Information Engineering Design Symposium*.

[71] Li J., Wang Y., Yan L. (2022). Novel Serological Biomarker Panel Using Protein Microarray Can Distinguish Active TB From Latent TB Infection. *Microbes and Infection*.

[72] Amrollahi F., Shashikumar S. P., Razmi F., Nemati S. (2020). Contextual Embeddings From Clinical Notes Improves Prediction of Sepsis. *AMIA Annu Symp Proc.*.

[73] Goh K. H., Wang L., Yeow A. Y. K. (2021). Artificial Intelligence in Sepsis Early Prediction and Diagnosis Using Unstructured Data in Healthcare. *Nature Communications*.

[74] Iqbal F., Chandra P., Lewis L. E. S. (2024). Application of Artificial Intelligence to Predict the Sepsis in Neonates Admitted in Neonatal Intensive Care Unit. *Journal of Neonatal Nursing*.

[75] Gao J., Lu Y., Ashrafi N., Domingo I., Alaei K., Pishgar M. (2024). Prediction of Sepsis Mortality in ICU Patients Using Machine Learning Methods. *BMC Medical Informatics and Decision Making*.

[76] Hu W. Multimodal Early Septic Shock Prediction Model Using Lasso Regression with Decaying Response.

[77] Yang M., Chen H., Hu W. (2024). Development and Validation of an Interpretable Conformal Predictor to Predict Sepsis Mortality Risk: Retrospective Cohort Study. *Journal of Medical Internet Research*.

[78] Liu F., Yao J., Liu C., Shou S. (2023). Construction and Validation of Machine Learning Models for Sepsis Prediction in Patients with Acute Pancreatitis. *BMC Surgery*.

[79] Hu C., Li L., Huang W. (2022). Interpretable Machine Learning for Early Prediction of Prognosis in Sepsis: a Discovery and Validation Study. *Infectious Disease and Therapy*.

[80] Misra D., Avula V., Wolk D. M. (2021). Early Detection of Septic Shock Onset Using Interpretable Machine Learners. *Journal of Clinical Medicine*.

[81] Wardi G., Carlile M., Holder A., Shashikumar S., Hayden S. R., Nemati S. (2021). Predicting Progression to Septic Shock in the Emergency Department Using an Externally Generalizable Machine-Learning Algorithm. *Annals of Emergency Medicine*.

[82] Wu M., Du X., Gu R., Wei J. (2021). Artificial Intelligence for Clinical Decision Support in Sepsis. *Frontiers of Medicine*.

[83] Sun C., Pei L. J., Zhang Y. L., Huang Y. G. (2022). [Deep Learning-Based Risk Prediction Model for Postoperative Healthcare-Associated Infections]. *Zhongguo Yi Xue Ke Xue Yuan Xue Bao*.

[84] Mao Q., Jay M., Hoffman J. L. (2018). Multicentre Validation of a Sepsis Prediction Algorithm Using Only Vital Sign Data in the Emergency Department, General Ward and ICU. *BMJ Open*.

[85] O’Reilly D., McGrath J., Martin-Loeches I. (2024). Optimizing Artificial Intelligence in Sepsis Management: Opportunities in the Present and Looking Closely to the Future. *J Intensive Med*.

[86] Pepic I., Feldt R., Ljungström L. (2021). Early Detection of Sepsis Using Artificial Intelligence: A Scoping Review Protocol. *Systematic Reviews*.

[87] Fleuren L. M., Klausch T. L. T., Zwager C. L. (2020). Machine Learning for the Prediction of Sepsis: A Systematic Review and Meta-Analysis of Diagnostic Test Accuracy. *Intensive Care Medicine*.

[88] Chen C. Y., Lin W. C., Yang H. Y. (2020). Diagnosis of Ventilator-Associated Pneumonia Using Electronic Nose Sensor Array Signals: Solutions to Improve the Application of Machine Learning in Respiratory Research. *Respiratory Research*.

[89] Rawson T. M., Hernandez B., Moore L. S. P. (2019). Supervised Machine Learning for the Prediction of Infection on Admission to Hospital: A Prospective Observational Cohort Study. *Journal of Antimicrobial Chemotherapy*.

[90] Mehrabi S., Krishnan A., Sohn S. (2015). DEEPEN: A Negation Detection System for Clinical Text Incorporating Dependency Relation into Negex. *Journal of Biomedical Informatics*.

[91] Bihorac A., Ozrazgat-Baslanti T., Ebadi A. (2019). Mysurgeryrisk: Development and Validation of a Machine-Learning Risk Algorithm for Major Complications and Death After Surgery. *Annals of Surgery*.

[92] Nachimuthu S. K., Haug P. J. (2012). Early Detection of Sepsis in the Emergency Department Using Dynamic Bayesian Networks. *AMIA*.

[93] Thottakkara P., Ozrazgat-Baslanti T., Hupf B. B. (2016). Application of Machine Learning Techniques to High-Dimensional Clinical Data to Forecast Postoperative Complications. *PLoS One*.

[94] Sendak M. P., Ratliff W., Sarro D. (2020). Real-World Integration of a Sepsis Deep Learning Technology Into Routine Clinical Care: Implementation Study. *JMIR Medical Informatics*.

[95] Valan B., Prakash A., Ratliff W. (2025). Evaluating Sepsis Watch Generalizability Through Multisite External Validation of a Sepsis Machine Learning Model. *npj Digital Medicine*.

[96] Boussina A., Shashikumar S. P., Malhotra A. (2024). Impact of a Deep Learning Sepsis Prediction Model on Quality of Care and Survival. *Digital Medicine*.

[97] Osheroff J. A., Teich J., Levick D. (2012). *Improving Outcomes with Clinical Decision Support: An Implementer’s Guide*.

[98] Berner E. S. (2007). *Clinical Decision Support Systems*.

[99] Sim I., Gorman P., Greenes R. A. (2001). Clinical Decision Support Systems for the Practice of Evidence-based Medicine. *Journal of the American Medical Informatics Association*.

[100] Omididan Z., Hadianfar A. (2011). The Role of Clinical Decision Support Systems in Healthcare (1980-2010): a Systematic Review Study. *Jentashapir Sceintific-Research Quarterly*.

[101] Shickel B., Tighe P. J., Bihorac A., Rashidi P. (2018). Deep EHR: a Survey of Recent Advances in Deep Learning Techniques for Electronic Health Record (EHR) Analysis. *IEEE journal of biomedical and health informatics*.

[102] Kwok R., Dinh M., Dinh D., Chu M. (2009). Improving Adherence to Asthma Clinical Guidelines and Discharge Documentation from Emergency Departments: Implementation of a Dynamic and Integrated Electronic Decision Support System. *Emergency Medicine Australasia*.

[103] Lipton J. A., Barendse R. J., Schinkel A. F., Akkerhuis K. M., Simoons M. L., Sijbrands E. J. (2011). Impact of an Alerting Clinical Decision Support System for Glucose Control on Protocol Compliance and Glycemic Control in the Intensive Cardiac Care Unit. *Diabetes Technology & Therapeutics*.

[104] Salem H., Caddeo G. (2018). A Multicentre Integration of a Computer-Led Follow-Up of Prostate Cancer is Valid and Safe. *BJU International*.

[105] Haberman S., Feldman J., Merhi Z. O., Markenson G., Cohen W., Minkoff H. (2009). Effect of Clinical-Decision Support on Documentation Compliance in an Electronic Medical Record. *Obstetrics & Gynecology*.

[106] Chen Z., Liang N., Zhang H. (2023). Harnessing the Power of Clinical Decision Support Systems: Challenges and Opportunities. *Open Heart*.

[107] Rajkomar A., Dean J., Kohane I. (2019). Machine Learning in Medicine. *New England Journal of Medicine*.

[108] Ferdush J., Begum M., Hossain S. T. (2024). Chatgpt and Clinical Decision Support: Scope, Application, and Limitations. *Annals of Biomedical Engineering*.

[109] Papadopoulos P., Soflano M., Chaudy Y., Adejo W., Connolly T. M. (2022). A Systematic Review of Technologies and Standards Used in the Development of Rule-Based Clinical Decision Support Systems. *Health Technology*.

[110] Elhaddad M., Hamam S. (2024). AI-Driven Clinical Decision Support Systems: An Ongoing Pursuit of Potential. *Cureus*.

[111] Rana M. S., Shuford J. (2024). AI in Healthcare: Transforming Patient Care Through Predictive Analytics and Decision Support Systems. *Journal of Artificial Intelligence General Science*.

[112] Obermeyer Z., Powers B., Vogeli C., Mullainathan S. (2019). Dissecting Racial Bias in an Algorithm Used to Manage the Health of Populations. *Science*.

[113] Olorunsogo T., Adenyi A. O., Okolo C. A., Babawarun O. (2024). Ethical Considerations in AI-enhanced Medical Decision Support Systems: A Review. *World Journal of Advanced Engineering Technology and Sciences*.

[114] Wan W., Xu J., Zeng Q., Pan L., Sun W. (2023). Development and Evaluation of Intelligent Medical Decision Support Systems. *Academic Journal of Science and Technology*.

[115] Boolchandani M., D’Souza A. W., Dantas G. (2019). Sequencing-Based Methods and Resources to Study Antimicrobial Resistance. *Nature Reviews Genetics*.

[116] Macesic N., Polubriaginof F., Tatonetti N. P. (2017). Machine Learning: Novel Bioinformatics Approaches for Combating Antimicrobial Resistance. *Current Opinion in Infectious Diseases*.

[117] Khaledi A., Schniederjans M., Pohl S. (2016). Transcriptome Profiling of Antimicrobial Resistance in *Pseudomonas aeruginosa*. *Antimicrobial Agents and Chemotherapy*.

[118] Nava Lara R. A., Aguilera-Mendoza L., Brizuela C. A., Peña A., Del Rio G. (2019). Heterologous Machine Learning for the Identification of Antimicrobial Activity in Human-Targeted Drugs. *Molecules*.

[119] Weinstein Z. B., Bender A., Cokol M. (2017). Prediction of Synergistic Drug Combinations. *Current Opinion in Systems Biology*.

[120] Rabaan A. A., Alhumaid S., Mutair A. A. (2022). Application of Artificial Intelligence in Combating High Antimicrobial Resistance Rates. *Antibiotics*.

[121] Olatunji I., Bardaji D. K. R., Miranda R. R., Savka M. A., Hudson A. O. (2024). Artificial Intelligence Tools for the Identification of Antibiotic Resistance Genes. *Frontiers in Microbiology*.

[122] Gupta A., Malwe A. S., Srivastava G. N., Thoudam P., Hibare K., Sharma V. K. (2022). MP4: a Machine Learning Based Classification Tool for Prediction and Functional Annotation of Pathogenic Proteins from Metagenomic and Genomic Datasets. *BMC Bioinformatics*.

[123] Arango-Argoty G., Garner E., Pruden A., Heath L. S., Vikesland P., Zhang L. (2018). Deeparg: A Deep Learning Approach for Predicting Antibiotic Resistance Genes from Metagenomic Data. *Microbiome*.

[124] Vihta K.-D., Pritchard E., Pouwels K. B. (2024). Predicting Future Hospital Antimicrobial Resistance Prevalence Using Machine Learning. *Communication and Medicine*.

[125] Gao Y., Li H., Zhao C., Li S., Yin G., Wang H. (2024). Machine Learning and Feature Extraction for Rapid Antimicrobial Resistance Prediction of Acinetobacter baumannii from whole-genome Sequencing Data. *Frontiers in Microbiology*.

[126] Fagiani F., Di Marino D., Romagnoli A. (2022). Molecular Regulations of Circadian Rhythm and Implications for Physiology and Diseases. *Signal Transduction and Targeted Therapy*.

[127] Diallo A. B., Coiffard B., Leone M., Mezouar S., Mege J.-L. (2020). For W.hom the Clock Ticks: Clinical Chronobiology for Infectious Diseases. *Frontiers in Immunology*.

[128] Kolben Y., Azmanov H., Gelman R., Dror D., Ilan Y. (2023). Using Chronobiology-based second-generation Artificial Intelligence Digital System for Overcoming Antimicrobial Drug Resistance in Chronic Infections. *Annals of Medicine*.

[129] Seymour C. W., Gesten F., Prescott H. C. (2017). Time to Treatment and Mortality During Mandated Emergency Care for Sepsis. *New England Journal of Medicine*.

[130] Liu V. X., Fielding-Singh V., Greene J. D. (2017). The Timing of Early Antibiotics and Hospital Mortality in Sepsis. *American Journal of Respiratory and Critical Care Medicine*.

[131] Taylor S. P., Anderson W. E., Beam K., Taylor B., Ellerman J., Kowalkowski M. A. (2021). The Association Between Antibiotic Delay Intervals and Hospital Mortality Among Patients Treated in the Emergency Department for Suspected Sepsis. *Critical Care Medicine*.

[132] Leisman D. E., Doerfler M. E., Ward M. F. (2017). Survival Benefit and Cost Savings from Compliance with a Simplified 3-hour Sepsis Bundle in a Series of Prospective, Multisite, Observational Cohorts. *Critical Care Medicine*.

[133] Hashemian S. M., Khoundabi B., Jamaati H. (2024). Correlation Between Cardiac Output and Disease Severity in Intubated COVID-19 Patients: Insights from Ultrasonic Cardiac Output Monitoring in Intensive Care Unit Settings. *Biomedical and Biotechnology Research Journal (BBRJ)*.

[134] Troncoso R., Garfinkel E. M., Hinson J. S., Smith A., Margolis A. M., Levy M. J. (2023). Do Prehospital Sepsis Alerts Decrease Time to Complete CMS Sepsis Measures?. *The American Journal of Emergency Medicine*.

[135] Leisman D. E., Deng H., Lee A. H. (2024). Effect of Automated Real-Time Feedback on Early-Sepsis Care: a Pragmatic Clinical Trial. *Critical Care Medicine*.

[136] Sawyer A. M., Deal E. N., Labelle A. J. (2011). Implementation of a real-time Computerized Sepsis Alert in Nonintensive Care Unit Patients. *Critical Care Medicine*.

[137] Hwang M. I., Bond W. F., Powell E. S. (2020). Sepsis Alerts in Emergency Departments: a Systematic Review of Accuracy and Quality Measure Impact. *Western Journal of Emergency Medicine*.

[138] Tarabichi Y., Cheng A., Bar-Shain D. (2022). Improving Timeliness of Antibiotic Administration Using a Provider and Pharmacist Facing Sepsis Early Warning System in the Emergency Department Setting: a Randomized Controlled Quality Improvement Initiative. *Critical Care Medicine*.

[139] Makam A. N., Nguyen O. K., Auerbach A. D. (2015). Diagnostic Accuracy and Effectiveness of Automated Electronic Sepsis Alert Systems: a Systematic Review. *Journal of Hospital Medicine*.

[140] Smyth M. A., Brace-McDonnell S. J., Perkins G. D. (2016). Identification of Adults with Sepsis in the Prehospital Environment: a Systematic Review. *BMJ Open*.

[141] Kelly C. J., Karthikesalingam A., Suleyman M., Corrado G., King D. (2019). Key Challenges for Delivering Clinical Impact with Artificial Intelligence. *BMC Medicine*.

[142] Menon K., Schlapbach L. J., Akech S. (2022). Criteria for Pediatric Sepsis-a Systematic Review and Meta-Analysis by the Pediatric Sepsis Definition Taskforce. *Critical Care Medicine*.

[143] Weiss S. L., Peters M. J., Alhazzani W. (2020). Surviving Sepsis Campaign International Guidelines for the Management of Septic Shock and Sepsis-Associated Organ Dysfunction in Children. *Intensive Care Medicine*.

[144] Habib A. R., Lin A. L., Grant R. W. (2021). The Epic Sepsis Model Falls Short—The Importance of External Validation. *JAMA Internal Medicine*.

[145] Kreitmann L., Bodinier M., Fleurie A. (2022). Mortality Prediction in Sepsis With an Immune-Related Transcriptomics Signature: A Multi-Cohort Analysis. *Frontiers of Medicine*.

[146] Aslan A. T., Permana B., Harris P. N., Naidoo K. D., Pienaar M. A., Irwin A. D. (2023). The Opportunities and Challenges for Artificial Intelligence to Improve Sepsis Outcomes in the Paediatric Intensive Care Unit. *Current Infectious Disease Reports*.

[147] Turakhia M. P., Desai M., Hedlin H. (2019). Rationale and Design of a Large-Scale, App-Based Study to Identify Cardiac Arrhythmias Using a Smartwatch: The Apple Heart Study. *American Heart Journal*.

[148] Davis S. E., Greevy R. A., Fonnesbeck C., Lasko T. A., Walsh C. G., Matheny M. E. (2019). A Nonparametric Updating Method to Correct Clinical Prediction Model Drift. *Journal of the American Medical Informatics Association*.

[149] Food A. D. (2019). Proposed Regulatory Framework for Modifications to Artificial intelligence/machine Learning (AI/ML)-based Software as a Medical Device (Samd).

[150] Wu L., Zhang J., Zhou W. (2019). Randomised Controlled Trial of WISENSE, a Real-Time Quality Improving System for Monitoring Blind Spots During Esophagogastroduodenoscopy. *Gut*.

[151] Wang P., Berzin T. M., Glissen Brown J. R. (2019). Real-Time Automatic Detection System Increases Colonoscopic Polyp and Adenoma Detection Rates: A Prospective Randomised Controlled Study. *Gut*.

[152] Vickers A. J., Cronin A. M., Elkin E. B., Gonen M. (2008). Extensions to Decision Curve Analysis, a Novel Method for Evaluating Diagnostic Tests, Prediction Models and Molecular Markers. *BMC Medical Informatics and Decision Making*.

[153] Gerke S., Babic B., Evgeniou T., Cohen I. G. (2020). The Need for a System View to Regulate Artificial Intelligence/Machine Learning-Based Software as Medical Device. *npj Digital Medicine*.

[154] (2006). *Building Explainable Artificial Intelligence Systems*.

[155] Nohara Y., Matsumoto K., Soejima H., Nakashima N. (2022). Explanation of Machine Learning Models Using Shapley Additive Explanation and Application for Real Data in Hospital. *Computer Methods and Programs in Biomedicine*.

[156] Scott H. F., Colborn K. L., Sevick C. J. (2020). Development and Validation of a Predictive Model of the Risk of Pediatric Septic Shock Using Data Known at the Time of Hospital Arrival. *The Journal of Pediatrics*.

[157] Bai Y. K. S., Kundu S., Askell A., Kernion J., Jones A. (2022). *Constitutional Ai: Harmlessness from Ai Feedback*.

[158] Imhoff M., Bauer M., Gather U., Löhlein D. (1998). Statistical Pattern Detection in Univariate Time Series of Intensive Care On-Line Monitoring Data. *Intensive Care Medicine*.

[159] Becker C., Gather U. (2001). The Largest Nonidentifiable Outlier: A Comparison of Multivariate Simultaneous Outlier Identification Rules. *Computational Statistics & Data Analysis*.

[160] Food U., Administration D. (2025). FDA Proposes Framework to Advance Credibility of AI Models Used for Drug and Biological Product Submissions.

